# Deep-learning time-series anomaly detection of acute kidney injury from creatinine–eGFR trajectories in the ICU

**DOI:** 10.1371/journal.pdig.0001411

**Published:** 2026-05-13

**Authors:** Yoonjin Kang, Soojeong Yun, Seung Min Song, Ji Eun Kim, Hyo Jin Kim, Eun Jung Cho, Shin Young Ahn, Young Joo Kwon, Min Woo Kang

**Affiliations:** 1 Department of Thoracic and Cardiovascular Surgery, Seoul National University Hospital, Seoul, Korea; 2 Seoul National University, College of Medicine, Seoul, Korea; 3 Department of Internal Medicine, Korea University Guro Hospital, Seoul, Korea; Oxford University: University of Oxford, UNITED KINGDOM OF GREAT BRITAIN AND NORTHERN IRELAND

## Abstract

Acute kidney injury (AKI) is common in the intensive care unit (ICU), and fixed creatinine thresholds may miss clinically relevant dynamics. We tested whether a deep-learning anomaly signal from short creatinine–estimated glomerular filtration rate (eGFR) series complements conventional criteria for risk stratification. Seven-step daily creatinine–eGFR instances were built from the Medical Information Mart for Intensive Care (MIMIC-III/IV; development/internal validation) and the eICU Collaborative Research Database (external validation). Time series began within 48 hours before ICU admission and ended at kidney replacement therapy (KRT), death, or ICU discharge. An unsupervised Anomaly Transformer trained on MIMIC produced final-step anomaly scores; the 95th percentile of training scores defined a fixed threshold. Anomaly-detected AKI required a final-step creatinine rise plus a score ≥ threshold. We compared scores across Kidney Disease: Improving Global Outcomes (KDIGO) stages and evaluated 24–96-hour KRT and mortality using area under the receiver operating characteristic curve (AUROC), accuracy, and F1-score. After exclusions, the internal dataset included 81,876 admissions (381,700 time-series instances) and the external dataset 140,237 admissions (494,684 instances). Anomaly scores increased stepwise across KDIGO categories and were higher in windows followed by KRT or death. For KRT prediction at 24, 48, 72, and 96 hours, AUROCs were 0.83, 0.82, 0.81, and 0.80 internally and 0.74 at all horizons externally. For mortality, AUROCs were 0.64–0.66 internally and 0.62–0.64 externally. In threshold-based classification, F1-scores were generally highest with the anomaly rule alone, whereas accuracy was greatest when requiring both anomaly detection and KDIGO stage ≥2. Event-capture analyses showed that anomaly detection identified more near-term KRT and mortality events than KDIGO stage ≥2, with the clearest separation for KRT. A creatinine–eGFR trajectory-based anomaly signal aligned with clinical severity, was associated with near-term outcomes, and appeared to complement KDIGO-based criteria in ICU populations.

## Author summary

We look after many critically ill patients whose kidneys suddenly become damaged. Doctors usually diagnose this “acute kidney injury” by asking whether a blood test called creatinine crosses fixed cut-off values. But in real ICU care, those rules can miss what we call “hidden acute kidney injury”: clinically consequential kidney injury that may not be flagged by fixed creatinine thresholds at the time of assessment because of baseline uncertainty, dilutional effects, or trajectory heterogeneity. In this study, we asked whether a computer model could uncover these hidden cases by watching how kidney tests change over time, rather than checking a single number. Using two large ICU databases, we fed the model short daily series of creatinine and an estimate of how well the kidneys filter blood. The model learned what typical trajectories look like and then gave each series an “anomaly” score that reflects how unusual it is. We found that higher anomaly scores were linked to a greater chance of starting dialysis or dying within the next few days, even when standard criteria were not met at the same assessment time point. Our results suggest that this trajectory-based signal could complement current definitions and help clinicians recognize hidden acute kidney injury.

## Introduction

Acute kidney injury (AKI) is common in critical care and is associated with substantial morbidity and mortality [1,2]. Contemporary Kidney Disease: Improving Global Outcomes (KDIGO) criteria provide a necessary common language by defining AKI through fixed changes in serum creatinine or urine output, yet uniform implementation in the intensive care unit (ICU) is challenging [3,4]. Challenges include uncertainty in the true baseline creatinine, dilutional effects from fluid accumulation that can mask injury, and rapidly evolving kidney function trajectories that do not conform to static thresholds [5–7]. These issues could shift incidence estimates and contribute to under-recognition in ICU populations, raising the possibility that clinically important kidney injury remains under-detected when diagnosis relies solely on static cutoffs.

Beyond whether a threshold is crossed, the shape of kidney function over time—its rise, fall, and fluctuation—conveys prognostic information that static maxima cannot capture. Multiple cohort studies show that distinct creatinine trajectories map to different risks and care pathways [7–10]. We refer to “hidden AKI” as kidney injury with near-term clinical consequences that may not be flagged by rule-based criteria at the time of assessment because of baseline uncertainty, dilutional effects, or non-monotonic dynamics.

Recent machine-learning work has largely focused on predicting future KDIGO-defined AKI from high-dimensional electronic records, demonstrating feasible lead times but mixed or null effects in randomized trials of electronic alerts on patient-centered outcomes [11–13]. This gap suggests that accurate detection of rule-defined events is not equivalent to identifying patients at imminent clinical risk. An alternative is a trajectory-aware, label-agnostic approach: learn typical physiologic dynamics directly from time series and flag atypical trajectories as candidate injury.

In this study, we operationalize a time-series anomaly signal computed from short daily trajectories of serum creatinine and estimated glomerular filtration rate (eGFR) [14]. We choose these two widely available measures to maximize portability across ICUs and minimize dependence on sparsely documented variables. Using an unsupervised transformer-based anomaly detector, we test whether the resulting signal aligns with clinical severity and captures near-term risk that complements KDIGO staging.

We prespecified three objectives. First, to assess whether anomaly scores increase monotonically across KDIGO AKI categories (face validity). Second, to determine whether higher anomaly scores are associated with kidney replacement therapy (KRT) initiation and mortality within 24–96 hours (clinical relevance). Third, to compare simple threshold-based classifications using the anomaly signal, KDIGO staging, and their combination in internal and external ICU datasets (complementarity and generalizability). By framing AKI as a trajectory problem rather than a threshold event, this work aims to provide a pragmatic, data-driven adjunct to current diagnostic practice in the ICU.

## Methods

### Ethical approval and consent to participate

This study was a secondary analysis of de-identified, publicly available data and was exempt from review by the Institutional Review Board of Seoul National University Hospital (No. 2405-061-1535). As all data are anonymized and freely accessible, individual informed consent was not required.

### Study setting and datasets

We conducted a retrospective analysis using two large, de-identified ICU databases. Model development and internal validation used Medical Information Mart for Intensive Care (MIMIC)-III and MIMIC-IV [15,16]. External validation used the eICU Collaborative Research Database (eICU-CRD) [17]. These databases were selected because they are large, publicly available ICU EHR resources that enable reproducible model development and an independent multi-center external validation, while providing the routinely collected variables. We identified ICU admissions with at least one kidney function measurement and required at least two measurements within the analytic window. We excluded admissions with KRT initiation or death within 24 hours of ICU admission and admissions with end-stage kidney disease. To support trajectory-based modeling and avoid reverse causation from immediate post-admission events, we applied these criteria to ensure sufficient longitudinal kidney-function data for window construction and to focus on incident near-term deterioration in non–dialysis-dependent patients. After applying these criteria, we converted each eligible record into time-series data and randomly split the MIMIC time-series into train (80%), validation (5%), and test (15%) sets. The held-out test set served as the prespecified internal validation data, and external validation on eICU-CRD was performed without model refitting or threshold recalibration.

### Construction of time-series features and outcome definitions

For each eligible admission, we assembled serum creatinine measurements and eGFR, with eGFR computed from creatinine, age, and sex using the 2021 Chronic Kidney Disease Epidemiology Collaboration creatinine-based race-free equation [18]. The series began at the first measurement obtained within 48 hours before ICU admission and ended at the earliest of KRT initiation, death, or ICU discharge. Measurements were aligned to a daily time grid by resampling at 24-hour intervals; days without observations were imputed by linear interpolation, a parsimonious baseline approach that is widely used and supported by recent benchmarking in health time-series imputation [19]. To reflect expected within-person variability and measurement uncertainty in serum creatinine values, we applied stochastic perturbation to creatinine values that were not directly observed, including both interpolated steps and left-padding steps, using literature-informed bounds (±11.4% for values ≥1 mg/dL and ±0.114 mg/dL for values <1 mg/dL) [20,21]. For left-padding, we repeated the earliest available measurement to prepend five leading steps and independently perturbed the padded creatinine at each step according to the same noise model. We selected a 7-day window (seven 24-hour steps) to align with the KDIGO creatinine-based AKI definition, which considers changes occurring within a 7-day interval. To create model inputs, we generated overlapping fixed-length windows in a rolling manner. Specifically, from the padded admission-level series we constructed seven-step instances (two variables: creatinine and eGFR) ending at each subsequent daily time point, such that the first instance contained five padded steps followed by the first two available patient steps, and subsequent instances were obtained by sliding the window forward one day at a time while retaining the seven most recent steps available up to that time point. Outcomes were defined relative to the final time point of each seven-step instance. For each instance, we created binary indicators for whether KRT initiation or death occurred within 24, 48, 72, or 96 hours after that final step. Each seven-step instance was treated as an independent observation for analysis.

### Model development and time-series anomaly–detected AKI

**Fig 1:**
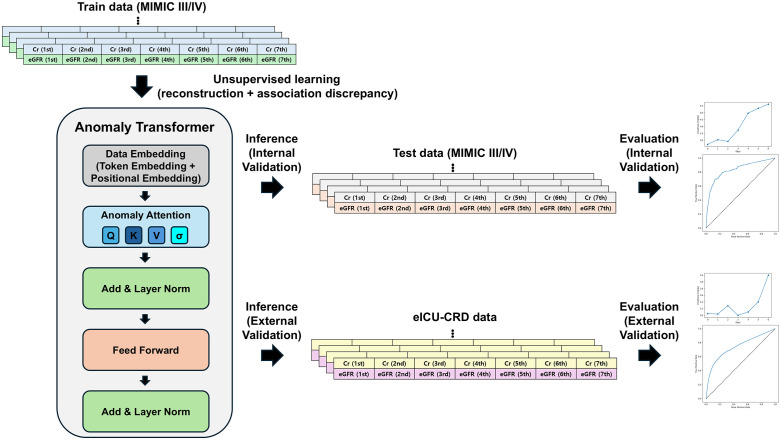
Analysis pipeline and anomaly transformer overview. Daily creatinine and eGFR were aligned to a 24-hour grid; missing days were imputed by linear interpolation with stochastic perturbation applied to non-observed creatinine steps. Admission-level series were left-padded and converted into rolling seven-step (7 × 24-hour) windows. The Anomaly Transformer was trained in MIMIC-III/IV; the final-step anomaly score $s_{T}$was evaluated using a fixed threshold $\tau_{\text{0}.\text{9}\text{5}}$(95th percentile of training $s_{T}$) and tested in internal and external validation without recalibration. Abbreviation: MIMIC, Medical Information Mart for Intensive Care; eICU-CRD, electronic Intensive Care Unit Collaborative Research Database; eGFR, estimated glomerular filtration.

We trained an unsupervised Anomaly Transformer for time-series anomaly detection using an association discrepancy objective on the train set of seven-step, 24-hour time-series windows of creatinine and eGFR [22]. Conceptually, the model learns what “typical” multi-day creatinine–eGFR trajectories look like by reconstructing the input sequence. It uses self-attention to learn which prior time points are most informative for reconstructing each step [23], while simultaneously imposing a simple distance-based temporal prior that favors nearby dependencies. When the learned attention pattern deviates from this prior and the sequence is harder to reconstruct, the resulting anomaly score increases. Because training is unsupervised, outcome labels are not required and are not used for threshold calibration, reducing the risk of label leakage and supporting portability. Early stopping was guided by the validation loss [24]. Details of the model configuration, optimization settings, and all hyperparameters are provided in the S1 Method. After training, the model produced an anomaly score at each time step, and we denote the final-step score by $s_{T}$. Using the train data, we calibrated a fixed decision threshold as the 95th percentile of $s_{T}$ ($\tau_{\text{0}.\text{9}\text{5}}$). We then applied this threshold in subsequent evaluations. We defined time-series anomaly–detected AKI when both conditions were satisfied: (1) the final-step creatinine increased ($c_{T}$*>*$c_{T-\text{1}}$), where $c_{T-\text{1}}$ and $c_{T}$ are the creatinine values at the last two 24-hour steps of the seven-step time-series window; and (2) the final-step anomaly score met or exceeded the threshold ($s_{T}$*≥*$\tau_{\text{0}.\text{9}\text{5}}$). A schematic of the architecture and analysis pipeline (training, inference, and evaluation) is provided in Fig 1. Outcome labels were not used during training or threshold calibration. Model development was conducted in Python (v3.11.7), and training was implemented in PyTorch (v2.3.1) on a system equipped with an AMD Ryzen 7 7800X3D CPU, 32 GB RAM, and an NVIDIA GeForce RTX 4070 Ti SUPER GPU (16 GB VRAM).

### Anomaly-score comparisons and predictive performance

We compared model-derived anomaly scores across AKI categories defined by KDIGO (no AKI, AKI stage 1, and AKI stage ≥2) and assessed group differences. AKI stage 1 was defined as a rise in serum creatinine of at least 0.3 mg/dL within 48 hours or an increase to 1.5 to <2.0 times the baseline within 7 days. AKI stage ≥2 was defined as an increase to at least 2.0 times the baseline. In line with the anomaly-detected rule, these comparisons used the subset with a final-step creatinine increase. We also compared anomaly scores across time-series windows stratified by whether each outcome (KRT or death) occurred within 24, 48, 72, or 96 hours after the window ended (after the final step), using two-sided Mann–Whitney U tests.

For outcome prediction, area under the receiver operating characteristics curve (AUROC) for KRT and mortality at 24, 48, 72, and 96 hours was computed on the subset with a final-step creatinine increase. Accuracy and F1-score were computed on the entire internal test dataset and the external eICU-CRD dataset for the following decision strategies [25]: (1) anomaly detection alone using the fixed threshold calibrated on the train set; (2) AKI stage 1; (3) AKI stage ≥2; and (4) both anomaly detection and AKI stage ≥2.

### Rule overlap, outcome capture, and risk-stratified association analyses

To characterize the complementarity between anomaly detection and KDIGO AKI stage ≥2 for identifying near-term adverse outcomes, we performed rule-overlap and risk-stratification analyses. First, we quantified outcome capture rates for KRT initiation and death within 24, 48, 72, and 96 hours after the final time point of each instance. For each horizon, capture was defined among outcome-positive instances as the proportion flagged at the final time point by (i) anomaly detection, (ii) KDIGO AKI stage ≥2, and (iii) either criterion. Capture rates were calculated at the window level using horizon-specific outcome-positive windows as the denominator and therefore should be interpreted as rule positivity among outcome-positive windows rather than as conventional patient-level sensitivity. Because rolling windows overlap within admissions, these values are descriptive window-level measures and are not directly comparable to patient-level sensitivity estimates. We further decomposed the either criterion into mutually exclusive categories—anomaly only (anomaly + /stage≥2−) and stage ≥2 only (anomaly − /stage≥2+)—to delineate which events were uniquely identified by each rule. Capture rates were summarized separately for the internal and external test sets and were reported for (a) all instances and (b) the subset with a final-step creatinine increase.

Second, we constructed four mutually exclusive risk strata based on the cross-classification of anomaly detection (positive/negative) and KDIGO AKI stage ≥2 (present/absent) at the final time point: anomaly − /stage≥2− (reference), anomaly + /stage≥2 − , anomaly − /stage≥2 + , and anomaly + /stage≥2 + . For each dataset and prediction horizon (24–96 hours), we estimated odds ratios (ORs) and 95% confidence intervals for outcome occurrence (KRT initiation or death) using logistic regression with risk stratum as the predictor and anomaly − /stage≥2− as the reference category.

### Selection and visualization of representative examples

To illustrate model behavior, we selected four representative instances using the last–time-point anomaly score. Examples were chosen to include both typical clinical AKI patterns defined by KDIGO and cases that did not meet KDIGO AKI thresholds but were detected by our model. Specifically, we present: (1) AKI stage ≥2 with anomaly detected and KRT within 48 hours; and (2) no AKI stage ≥2 but anomaly detected with KRT within 48 hours.

### Sensitivity analyses

We performed sensitivity analyses to evaluate the robustness of predictive performance to missing-data handling, anomaly-score threshold specification, and effective input window length. First, we repeated the AUROC analyses for predicting KRT initiation and death using only admissions with complete creatinine time series across the analytic period. As in the primary analysis, AUROC was computed using the subset of instances that exhibited an increase in creatinine between the final two time points. Second, we evaluated the sensitivity of threshold-based classification performance to the anomaly-score cutoff by redefining the decision threshold using alternative upper-tail percentiles of the anomaly-score distribution in the training set (1%, 2.5%, and 10%). For each threshold definition, we recalculated accuracy, F1-score, precision, and recall for predicting KRT initiation and death at prediction horizons of 24, 48, 72, and 96 hours. Third, we examined sensitivity to the amount of historical information available by varying the effective input window length from 2 to 6 days. For each setting, we constructed model inputs by applying simple left-padding to reach the fixed seven-step format, repeating the earliest available measurement within an instance to fill the leading steps. We then recomputed AUROC for KRT initiation and death at each prediction horizon, again restricting evaluation to instances with a final-step creatinine increase.

## Results

### Study population and baseline characteristics

**Fig 2:**
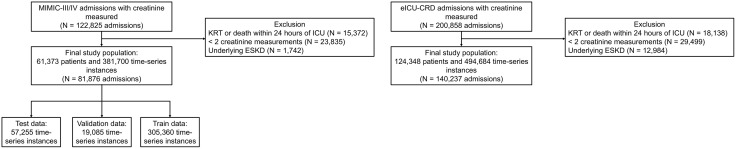
Study population flow diagram. Selection and exclusions for MIMIC-III/IV (development/internal validation) and eICU-CRD (external validation), including exclusion of admissions with ESKD and those with KRT initiation or death within 24 hours of ICU admission. Final numbers of admissions and derived windows are shown. Abbreviation: MIMIC, Medical Information Mart for Intensive Care; KRT, kidney replacement therapy; ICU, intensive care unit; ESKD, end-stage kidney disease; eICU-CRD, electronic Intensive Care Unit Collaborative Research Database.

Model development and internal validation used MIMIC-III and MIMIC-IV. In these datasets, there were 81,876 admissions from 61,373 patients, yielding 381,700 time-series instances (train: 305,360; validation: 19,085; test: 57,255). External validation used the eICU-CRD. In this dataset, there were 140,237 admissions from 124,348 patients, yielding 494,684 time-series instances. Participant flow and dataset partitioning are summarized in Fig 2.

Baseline characteristics are summarized in Table 1. In MIMIC-III/IV, males constituted 56.3% and mean age was 66.38 years. The mean of the lowest creatinine values was 0.97 mg/dL and the mean of the highest creatinine values was 1.51 mg/dL; the corresponding means for eGFR were 64.65 and 87.19 mL/min/1.73 m². KRT prior to discharge occurred in 3.0%, and mortality was 6.9%. In eICU-CRD, males constituted 53.9% and mean age was 63.49 years. The lowest and highest creatinine values were 1.02 mg/dL and 1.60 mg/dL, and the lowest and highest eGFR values were 63.48 and 86.26 mL/min/1.73 m². KRT prior to discharge occurred in 1.8%, and mortality was 8.0%.

**Table 1:** Baseline characteristics.

Variable	MIMIC-III and IV (N = 81876)	eICU-CRD (N = 140237)
**Male**	46124 (56.3%)	75572 (53.9%)
**Age (years)**	66.38 (30.09)	63.49 (17.08)
**Lowest creatinine (mg/dL)**	0.97 (0.80)	1.02 (0.90)
**Highest creatinine (mg/dL)**	1.51 (1.26)	1.60 (1.50)
**Lowest eGFR (mL/min/1.73m**^**2**^)	64.65 (31.86)	63.48 (31.42)
**Highest eGFR (mL/min/1.73m**^**2**^)	87.19 (32.96)	86.26 (32.06)
**KRT before discharge**	2430 (3.0%)	2524 (1.8%)
**In-hospital mortality**	5631 (6.9%)	11287 (8.0%)

Values are presented as n (%) for categorical variables and mean (standard deviation) for continuous variables.

Abbreviation: MIMIC, Medical Information Mart for Intensive Care; eICU-CRD, electronic Intensive Care Unit Collaborative Research Database; eGFR, estimated glomerular filtration rate; KRT, kidney replacement therapy.

### Data interpolation and anomaly transformer training

Across the assembled daily series, 10.74% of 24-hour steps were imputed by linear interpolation. At the admission level, missingness was generally sparse but common: the mean number of missing creatinine days per admission was 0.61 in MIMIC-III/IV and 0.55 in eICU-CRD, and 50.27% and 37.65% of admissions, respectively, had at least one missing day; most admissions had no missing days or only one missing day (S1 and S2 Tables). Baseline characteristics were largely comparable by missingness status for sex and age, whereas creatinine distributions and outcome rates differed, including substantially lower KRT before discharge among admissions with missing days (S3 Table). The analysis workflow and model pipeline (training on MIMIC, internal/external inference, evaluation) are summarized in Fig 1. The 95th-percentile anomaly threshold derived from the train set was 0.131 and was held fixed without recalibration for all subsequent analyses.

### Anomaly-score comparisons

**Fig 3:**
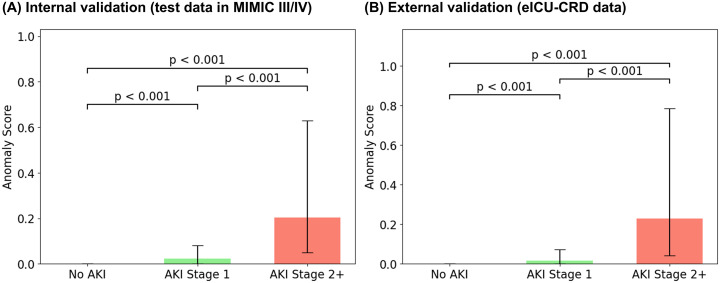
Anomaly scores by acute kidney injury stage. Final-step anomaly scores $s_{T}$ across AKI categories (no AKI, stage 1, stage ≥2) in MIMIC-III/IV (test) and eICU-CRD. Abbreviation: MIMIC, Medical Information Mart for Intensive Care; eICU-CRD, electronic Intensive Care Unit Collaborative Research Database; AKI, acute kidney injury.

**Fig 4:**
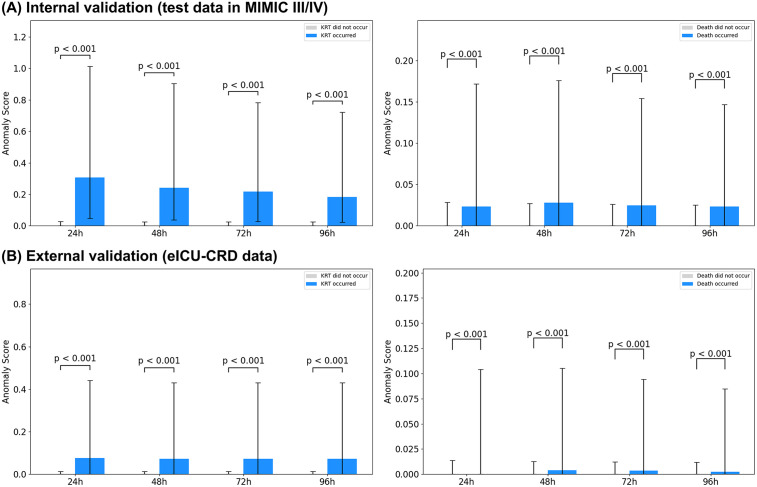
Anomaly scores by near-term outcomes. Final-step anomaly scores $s_{T}$ stratified by KRT initiation or in-hospital mortality within 24–96 hours after the window end, shown for MIMIC-III/IV (test) and eICU-CRD. Abbreviation: MIMIC, Medical Information Mart for Intensive Care; eICU-CRD, electronic Intensive Care Unit Collaborative Research Database; KRT, kidney replacement therapy.

Anomaly scores increased in a stepwise fashion across KDIGO-defined AKI categories (no AKI, stage 1, stage ≥2). In both the internal development dataset (MIMIC-III/IV) and the external dataset (eICU-CRD), scores were significantly higher for AKI stage 1 and AKI stage ≥2 than for no AKI, and significantly higher for AKI stage ≥2 than for AKI stage 1 (Fig 3). When stratified by outcome occurrence within 24, 48, 72, or 96 hours, anomaly scores were consistently higher in the event-positive groups for KRT and mortality across both datasets (Fig 4). Across horizons, scores in the KRT-positive groups generally exceeded those in the mortality-positive groups.

### Evaluation of predictive performance

**Fig 5:**
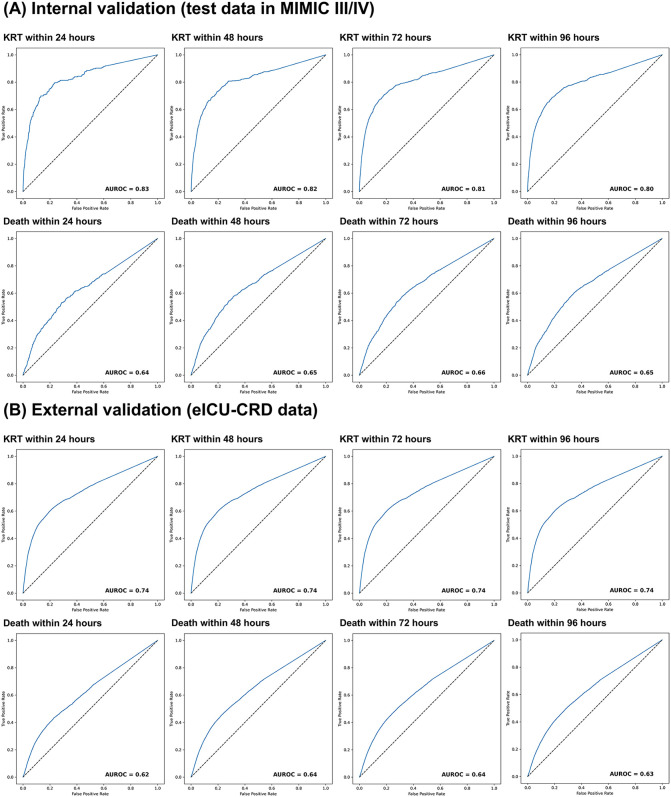
Receiver operating characteristic curves for predicting near-term outcomes by the anomaly score. ROC curves and AUROCs for predicting KRT initiation and in-hospital mortality within 24–96 hours after the window end using the continuous final-step anomaly score $s_{T}$, shown for MIMIC-III/IV (test) and eICU-CRD. Evaluation used windows with a last-step creatinine increase. Abbreviation: MIMIC, Medical Information Mart for Intensive Care; eICU-CRD, electronic Intensive Care Unit Collaborative Research Database; KRT, kidney replacement therapy; ROC, receiver operating characteristic curve; AUROC, area under the receiver operating characteristic curve.

Receiver operating characteristic (ROC) curves for predicting KRT and mortality within 24, 48, 72, and 96 hours using the final-step anomaly score are shown in Fig 5. In internal validation, AUROCs for KRT were 0.83, 0.82, 0.81, and 0.80 at 24, 48, 72, and 96 hours, respectively; for mortality they were 0.64, 0.65, 0.66, and 0.65. In external validation, AUROC for KRT was 0.74 at all horizons, and for mortality 0.62, 0.64, 0.64, and 0.63 at 24, 48, 72, and 96 hours, respectively.

In threshold-based classification, across datasets and outcomes, F1-scores were generally highest with the anomaly-detection rule alone, whereas accuracy was maximized when classification required concurrence of anomaly detection and AKI stage ≥2. At the 48-hour horizon, KRT prediction in the internal dataset achieved the highest accuracy of 0.98 with the combined criterion, while the highest F1 was 0.20 with anomaly detection alone (Table 2). In the external dataset, accuracy for KRT was 0.97 with the combined criterion, and the highest F1 was 0.16 with anomaly detection alone. For mortality within 48 hours, the internal dataset reached an accuracy of 0.96 with the combined criterion and an F1 of 0.13 with anomaly detection alone; in the external dataset, accuracy was 0.96 with the combined criterion, and the highest F1 was 0.13 with AKI stage ≥2 alone (Table 3).

**Table 2:** Comparison of performance metrics for predicting kidney replacement therapy.

Dataset	Outcome	Method	Accuracy	F1 score
Internal validation (test data in MIMIC III/IV)				
	KRT within 24 hours	AKI stage 1	0.81	0.05
		AKI stage 2+	0.96	0.10
		Anomaly Transformer	0.96	0.11
		Anomaly Transformer and AKI stage 2+	**0.98**	**0.13**
	KRT within 48 hours	AKI stage 1	0.82	0.09
		AKI stage 2+	0.96	0.16
		Anomaly Transformer	0.96	**0.20**
		Anomaly Transformer and AKI stage 2+	**0.98**	**0.20**
	KRT within 72 hours	AKI stage 1	0.82	0.13
		AKI stage 2+	0.96	0.19
		Anomaly Transformer	0.96	**0.25**
		Anomaly Transformer and AKI stage 2+	**0.97**	0.23
	KRT within 96 hours	AKI stage 1	0.82	0.14
		AKI stage 2+	0.95	0.21
		Anomaly Transformer	0.96	**0.26**
		Anomaly Transformer and AKI stage 2+	**0.97**	0.24
External validation (eICU-CRD)				
	KRT within 24 hours	AKI stage 1	0.84	0.11
		AKI stage 2+	0.96	0.13
		Anomaly Transformer	0.96	**0.15**
		Anomaly Transformer and AKI stage 2+	**0.97**	0.13
	KRT within 48 hours	AKI stage 1	0.84	0.11
		AKI stage 2+	0.96	0.13
		Anomaly Transformer	0.96	**0.16**
		Anomaly Transformer and AKI stage 2+	**0.97**	0.13
	KRT within 72 hours	AKI stage 1	0.84	0.11
		AKI stage 2+	0.96	0.13
		Anomaly Transformer	0.96	**0.16**
		Anomaly Transformer and AKI stage 2+	**0.97**	0.13
	KRT within 96 hours	AKI stage 1	0.84	0.11
		AKI stage 2+	0.96	0.13
		Anomaly Transformer	0.96	**0.16**
		Anomaly Transformer and AKI stage 2+	**0.97**	0.13

Abbreviation: MIMIC, Medical Information Mart for Intensive Care; KRT, kidney replacement therapy; AKI, acute kidney injury; eICU-CRD, electronic Intensive Care Unit Collaborative Research Database.

**Table 3:** Comparison of performance metrics for predicting in-hospital mortality.

Dataset	Outcome	Method	Accuracy	F1 score
Internal validation (test data in MIMIC III/IV)				
	Death within 24 hours	AKI stage 1	0.81	0.07
		AKI stage 2+	0.95	**0.08**
		Anomaly Transformer	0.95	**0.08**
		Anomaly Transformer and AKI stage 2+	**0.97**	0.07
	Death within 48 hours	AKI stage 1	0.81	0.11
		AKI stage 2+	0.95	0.12
		Anomaly Transformer	0.94	**0.13**
		Anomaly Transformer and AKI stage 2+	**0.96**	0.10
	Death within 72 hours	AKI stage 1	0.81	0.15
		AKI stage 2+	0.94	**0.14**
		Anomaly Transformer	0.94	**0.14**
		Anomaly Transformer and AKI stage 2+	**0.95**	0.10
	Death within 96 hours	AKI stage 1	0.81	**0.18**
		AKI stage 2+	0.93	0.15
		Anomaly Transformer	0.93	0.15
		Anomaly Transformer and AKI stage 2+	**0.95**	0.11
External validation (eICU-CRD)				
	Death within 24 hours	AKI stage 1	0.83	0.07
		AKI stage 2+	0.96	**0.09**
		Anomaly Transformer	0.96	0.07
		Anomaly Transformer and AKI stage 2+	**0.97**	0.07
	Death within 48 hours	AKI stage 1	0.83	0.12
		AKI stage 2+	0.95	**0.13**
		Anomaly Transformer	0.95	0.11
		Anomaly Transformer and AKI stage 2+	**0.96**	0.09
	Death within 72 hours	AKI stage 1	0.83	**0.16**
		AKI stage 2+	0.94	0.14
		Anomaly Transformer	0.94	0.13
		Anomaly Transformer and AKI stage 2+	**0.95**	0.09
	Death within 96 hours	AKI stage 1	0.83	**0.19**
		AKI stage 2+	0.93	0.14
		Anomaly Transformer	0.93	0.13
		Anomaly Transformer and AKI stage 2+	**0.94**	0.09

Abbreviation: MIMIC, Medical Information Mart for Intensive Care; AKI, acute kidney injury; eICU-CRD, electronic Intensive Care Unit Collaborative Research Database.

### Outcome capture and risk stratification by anomaly detection and KDIGO AKI stage ≥2

Across both datasets and all prediction horizons, anomaly detection captured a larger proportion of near-term events than AKI stage ≥2, and this difference was more pronounced in windows with a final-step creatinine increase. For KRT, anomaly detection captured approximately half of events in internal validation across all windows (46.6–49.2%) compared with 30.6–34.2% for AKI stage ≥2; the separation widened further in last-time-point creatinine-rising windows (57.4–62.7% vs 38.1–44.1%) (Table 4). External validation showed lower absolute capture in all windows, yet the same directional pattern and a clearer separation in creatinine-rising windows, where anomaly detection captured 47.7–53.9% of KRT events versus 27.1–29.5% for AKI stage ≥2. Decomposition of uniquely captured events demonstrated that “anomaly only” consistently exceeded “AKI stage ≥2 only,” particularly in creatinine-rising windows, indicating that anomaly detection contributed additional KRT event capture beyond AKI stage ≥2 in both internal and external validation. For in-hospital mortality, overall capture rates were lower than for KRT, but anomaly detection again generally exceeded AKI stage ≥2 across horizons in both datasets, with the advantage persisting in creatinine-rising windows (Table 5).

**Table 4:** Event capture rates for kidney replacement therapy.

Dataset	Outcome time horizon (hours)	All windows					Last-time-point creatinine rising windows				
		Anomaly captured	AKI stage2 captured	Either captured	Anomaly only	AKI stage2 only	Anomaly captured	AKI stage2 captured	Either captured	Anomaly only	AKI stage2 only
Internal validation (test data in MIMIC III/IV)	24	49.2%	34.2%	53.6%	19.4%	4.4%	62.7%	44.1%	67.8%	23.7%	5.1%
	48	48.1%	32.3%	52.8%	20.7%	4.8%	59.1%	40.3%	64.7%	24.6%	5.8%
	72	48.6%	30.9%	53.6%	23.3%	5.5%	59.2%	38.1%	65.1%	27.5%	6.5%
	96	46.6%	30.6%	51.8%	21.9%	5.9%	57.4%	38.3%	63.8%	26.1%	7.2%
External validation (eICU-CRD)	24	23.7%	14.3%	25.7%	11.3%	2.0%	47.7%	27.1%	50.7%	23.6%	3.0%
	48	34.0%	18.2%	35.8%	20.4%	3.4%	52.3%	27.7%	55.3%	29.8%	4.3%
	72	36.4%	19.3%	38.3%	23.0%	5.0%	53.0%	28.7%	56.3%	31.5%	6.4%
	96	37.7%	20.3%	39.6%	24.5%	5.7%	53.9%	29.5%	57.3%	33.2%	7.3%

Abbreviation: AKI, acute kidney injury; MIMIC, Medical Information Mart for Intensive Care; eICU-CRD, electronic Intensive Care Unit Collaborative Research Database.

Capture rates were calculated separately at each horizon using horizon-specific outcome-positive windows as the denominator and therefore should not be interpreted as cumulative patient-level sensitivity.

**Table 5:** Event capture rates for in-hospital mortality.

Dataset	Outcome time horizon (hours)	All windows					Last-time-point creatinine rising windows				
		Anomaly captured	AKI stage2 captured	Either captured	Anomaly only	AKI stage2 only	Anomaly captured	AKI stage2 captured	Either captured	Anomaly only	AKI stage2 only
Internal validation (test data in MIMIC III/IV)	24	20.4%	16.5%	26.7%	10.3%	6.3%	28.2%	25.5%	38.0%	12.4%	9.7%
	48	20.1%	15.8%	25.6%	9.8%	5.9%	27.8%	24.3%	36.3%	12.0%	9.0%
	72	19.8%	15.5%	24.9%	9.5%	5.7%	26.9%	23.3%	34.6%	11.6%	8.6%
	96	19.1%	15.2%	24.1%	9.2%	5.8%	26.6%	23.3%	34.5%	11.6%	8.8%
External validation (eICU-CRD)	24	17.9%	15.3%	22.8%	7.6%	4.9%	24.4%	20.9%	31.0%	10.2%	6.7%
	48	20.5%	15.6%	24.9%	10.6%	6.7%	24.8%	19.9%	30.9%	12.1%	7.9%
	72	21.6%	15.9%	25.7%	12.1%	7.4%	25.0%	19.3%	30.5%	13.3%	8.4%
	96	22.0%	16.2%	26.2%	12.8%	7.9%	24.9%	19.2%	30.5%	13.7%	8.9%

Abbreviation: AKI, acute kidney injury; MIMIC, Medical Information Mart for Intensive Care; eICU-CRD, electronic Intensive Care Unit Collaborative Research Database.

Capture rates were calculated separately at each horizon using horizon-specific outcome-positive windows as the denominator and therefore should not be interpreted as cumulative patient-level sensitivity.

Risk-stratified analyses similarly demonstrated graded increases in near-term risk across strata defined by anomaly detection and AKI stage ≥2. For both outcomes, each non-reference stratum (anomaly + /stage≥2 − , anomaly − /stage≥2 + , and anomaly + /stage≥2+) showed substantially higher event rates and significantly elevated odds relative to anomaly − /stage≥2− across all horizons in both datasets (all p < 0.001). For KRT, the anomaly + /stage≥2 − stratum consistently exhibited higher ORs than the stage≥2-only stratum in both internal and external validation across horizons, indicating stronger risk enrichment by anomaly positivity than by AKI stage ≥2 (S4 Table). As expected, the highest risk occurred when both criteria were present, with the largest ORs at every horizon in both datasets. For mortality, ORs were also consistently elevated for the anomaly-only, stage≥2-only, and combined strata, with the combined stratum showing the greatest risk (S5 Table).

### Examples of anomaly detection

**Fig 6:**
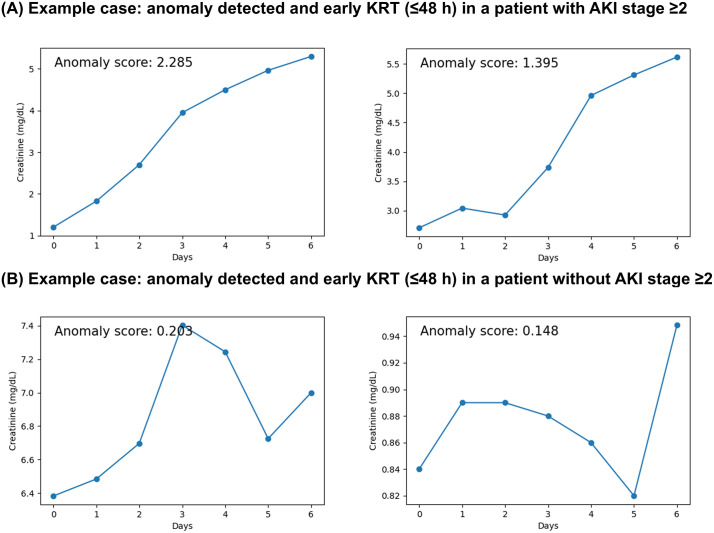
Representative cases of anomaly detection. Examples illustrating creatinine trajectories and corresponding anomaly scores $s_{T}$ with rule status (anomaly threshold $\tau_{\text{0}.\text{9}\text{5}}$, AKI stage ≥2) and near-term KRT occurrence. Abbreviation: KRT, kidney replacement therapy; AKI, acute kidney injury.

Fig 6 presents four representative instances with paired visualizations: an AKI stage ≥2 case with anomaly detection and KRT within 48 hours; a case without AKI stage ≥2 in which the model detected an anomaly and KRT was initiated within 48 hours; an AKI stage ≥2 case without anomaly detection and without KRT within 48 hours; and a case without AKI stage ≥2 without anomaly detection and without KRT within 48 hours. Notably, some patients who did not meet KDIGO stage ≥2 were nevertheless identified as anomalous by the model and underwent KRT within 48 hours. In the anomaly-detected instance with AKI stage ≥2 and KRT within 48 hours, creatinine increased persistently across the seven 24-hour steps and the final-step anomaly score was high. In the anomaly-detected instance without AKI stage ≥2 and with KRT within 48 hours, creatinine exhibited marked fluctuations rather than a sustained rise, and the final-step anomaly score was lower than in the stage ≥2 example.

### Sensitivity analyses

Sensitivity analyses were consistent with the primary findings. In complete-case admissions, discrimination was preserved, with AUROC for KRT ranging from 0.78 to 0.81 in internal validation and remaining 0.73 across horizons in external validation; mortality AUROC ranged from 0.66 to 0.73 internally and from 0.65 to 0.70 externally (S6 Table). Threshold variation demonstrated the anticipated precision–recall trade-off, whereby more stringent anomaly-score cutoffs maintained high accuracy but reduced recall, while less stringent cutoffs increased recall at the expense of accuracy and precision; intermediate thresholds generally yielded the highest F1-scores for KRT, with similar qualitative patterns for mortality (S7 and S8 Tables). Performance was also strongly dependent on the amount of longitudinal information, as shorter input windows substantially reduced AUROC; however, windows of 3–4 days still achieved modest discrimination, and windows of 5–6 days approached the primary performance in both datasets (S9 Table).

## Discussion

Across two large ICU datasets, a deep learning–based time-series anomaly score derived from daily creatinine and eGFR was consistently higher among patients who underwent KRT or died within 24–96 hours, and it discriminated these outcomes over the same horizons. Discrimination varied by outcome, with AUROC overall modest in absolute terms but generally higher for KRT than for death, which is expected because the model is unsupervised and relies solely on creatinine and eGFR trajectories. While this trajectory-based signal has more limited utility for predicting death, it appears more useful for identifying near-term KRT risk and can complement KDIGO staging as an indicator of impending kidney-related deterioration. In comparative analyses of anomaly-detected AKI versus AKI defined by KDIGO, the anomaly-based rule generally achieved the highest F1-scores across datasets and horizons. This pattern supports functional complementarity rather than replacement. Event-capture analyses aligned with this interpretation. Across internal and external validation, anomaly detection captured a larger proportion of near-term events than KDIGO AKI stage ≥2, and the anomaly-only contribution exceeded the stage≥2-only contribution. This separation was most pronounced for KRT and was also observed for mortality across horizons. Joint risk stratification further revealed marked gradients in near-term risk, and for KRT the anomaly-positive/stage≥2-negative stratum generally showed higher ORs than the anomaly-negative/stage≥2-positive stratum in both datasets, indicating that anomaly positivity enriched near-term KRT risk at least as strongly as KDIGO stage ≥2 when considered in isolation. Taken together, these findings indicate that a trajectory-aware, model-derived signal can complement the conventional KDIGO definition by capturing kidney dynamics that are closely linked to imminent clinical deterioration.

Clinically, the proposed anomaly score is best viewed as a trajectory-based adjunct to KDIGO staging rather than a replacement. Because it can be recalculated at each daily update from routinely available creatinine, it could be integrated into the electronic medical record (EMR) and displayed alongside KDIGO stage as a complementary “instability” signal reflecting recent kidney dynamics. When the anomaly signal is positive even when conventional KDIGO thresholds are not met at the time of assessment, it may prompt focused clinical reassessment of potentially modifiable contributors to kidney instability, such as nephrotoxic medication exposures, hemodynamic and volume status, and other reversible causes of kidney injury. In practice, it could support EMR-based triage in large centers and facilitate prioritized tele-nephrology review or escalation pathways in smaller or remote settings where dialysis resources are limited. Conversely, requiring concurrence of anomaly positivity and KDIGO AKI stage ≥2 can serve as a higher-specificity operating point to reduce alert burden. Pilot implementation should include site-level calibration of the operating threshold, predefined alert-routing pathways, and prospective evaluation of both alert burden and clinical utility.

This trajectory-centric approach contrasts with prior machine-learning work that predicted future rule-defined AKI from high-dimensional electronic records. Rather than optimizing for threshold crossings, we employ label-agnostic, deep learning–based time-series anomaly detection to model kidney trajectories and then relate the resulting signal to hard clinical endpoints, including KRT and mortality. In this framework, differences in performance across 24–96 hour horizons should not be interpreted as evidence of superior longer-range prediction alone, because they may partly reflect increasing temporal proximity to the outcome at the time of evaluation. AKI is therefore treated here as a problem of unstable kidney dynamics rather than a single-event threshold, allowing the model to surface atypical patterns even when fixed cutoffs are not met at the time of assessment. Evidence from randomized trials indicates that electronic AKI alerts can improve recognition yet do not consistently improve patient-centered outcomes, underscoring that static thresholds alone may be insufficient for actionable phenotyping in the ICU [12,13]. By design, a trajectory-aware anomaly signal is readily composable with existing alerting systems, which may help target scarce nephrology and dialysis resources to patients at highest near-term risk.

A key conceptual contribution is the delineation of hidden AKI, defined as clinically consequential kidney injury that may be underdetected when diagnosis is operationalized solely by static cutoffs [26–29]. In critical illness, baseline creatinine is frequently indeterminate, which complicates change-based rules [30]. In addition, heterogeneity in creatinine trajectories, including rapid monotonic rises, oscillatory patterns, and incomplete recoveries, conveys prognostic information that static maxima do not capture [10,31]. Furthermore, fluid accumulation and the delayed kinetics of creatinine generation and distribution can obscure injury at the time of assessment, making trajectory-sensitive methods attractive for near-term risk identification in the ICU. Taken together, these considerations support the use of a trajectory-aware anomaly signal to surface occult patterns associated with adverse outcomes even when conventional AKI criteria are not yet met.

Methodologically, the Anomaly Transformer suits ICU time series for three reasons. First, it combines self-attention with a simple proximity prior that favors relationships between nearby time points, which is well matched to ICU creatinine trajectories where the most informative changes often occur over adjacent days [22]. At the same time, it still permits longer-range dependencies when supported by the data, allowing the model to relate current kidney function to earlier baseline levels or slower-moving trends across the observation window. Second, it learns a discrepancy between data-driven associations and the prior, yielding a per-time-step anomaly signal that functions as a soft change-point detector in an unsupervised setting [14]. Third, it produces stepwise scores that can be aligned to the final evaluation step and visualized over time to localize when a kidney trajectory becomes atypical. We considered alternative families of time-series anomaly detection methods, including reconstruction- and density-based approaches (e.g., autoencoder or variational autoencoder variants) as well as other neural time-series anomaly detectors commonly used in the literature [32,33]. We selected the Anomaly Transformer because its anomaly score is explicitly grounded in the mismatch between learned temporal associations and a simple distance-based prior, which is well aligned with short, clinically sampled trajectories and provides an interpretable, time-resolved signal rather than a single global reconstruction error [22,32]. In addition, the minimal two-variable design (creatinine and eGFR) favors deployability across heterogeneous ICUs, where richer covariates (e.g., urine output) are often missing or inconsistently recorded. These properties match our design of seven-step daily windows and support a simple fixed-quantile thresholding rule for downstream decisions.

Sensitivity analyses further indicated that the anomaly threshold functions as a tunable clinical operating point. Varying the cutoff produced the expected precision–recall trade-off across datasets and horizons, supporting threshold selection based on institutional tolerance for false positives and alert burden rather than reliance on a single fixed value. In this context, modest absolute F1-scores are expected because near-term KRT and mortality are relatively rare outcomes and the anomaly threshold was prespecified (rather than optimized for F1), making operating-point metrics particularly sensitive to outcome imbalance. In addition, shortening the effective input window substantially reduced discrimination; in particular, 2-day windows performed poorly, whereas 3–4-day windows retained modest performance and 5–6-day windows approached the primary performance, providing convergent evidence that the model leverages recent multi-day creatinine dynamics rather than isolated measurements. Taken together, these findings suggest that the approach may be better suited to ongoing inpatient risk monitoring after several days of observed kidney-function data than to very early ICU triage or short admissions.

Limitations include the restricted feature set, as we modeled creatinine and eGFR resampled at 24-hour intervals with interpolation, and the exclusion of urine output and other covariates because of substantial missingness and documentation inaccuracy in the ICU. Creatinine missingness at the daily level was generally sparse but common at the admission level, and it was not fully random. Admissions with missing creatinine days were similar in age and sex but tended to exhibit lower creatinine values and lower rates of KRT, suggesting that observation patterns may correlate with underlying acuity and care processes. Accordingly, we complemented the primary analyses with complete-case and sensitivity analyses to evaluate robustness to missingness and preprocessing choices, and discrimination was preserved in complete-case admissions, indicating that the primary findings were not driven solely by interpolation or missingness patterns. Measurement frequency may itself correlate with clinical severity; although our design focused on fixed-interval resampling, future work should explicitly account for observation density as a potential confounder. In addition, KRT was ascertained from charting, procedure, and order-derived records, and imperfect cross-database alignment of these variables may have introduced limited outcome misclassification. Moreover, cause-specific drivers of death were not consistently available, limiting mechanistic interpretation of the mortality analyses. Furthermore, because orthogonal biomarkers of kidney damage were unavailable, the anomaly signal may partly reflect non-kidney influences on creatinine trajectories, such as changes in volume status or creatinine generation, which limits biological specificity. Most importantly, although we identified AKI using an anomaly-detected definition and demonstrated associations with clinical outcomes, we could not verify underlying kidney injury biology with orthogonal damage measures or histopathology, so validation relied on clinical outcomes as surrogates. Prospective studies that integrate damage biomarkers and embed a trajectory-aware anomaly signal into real-time workflows are needed to determine whether this approach changes care processes and improves outcomes.

A time-series anomaly–detected definition of AKI based on creatinine and eGFR trajectories appears to align with clinical severity, was associated with near-term KRT and mortality within 24–96 hours, and may complement KDIGO-based diagnostic criteria. By reframing AKI as a trajectory phenomenon and providing a portable, label-agnostic signal, this work offers a pragmatic adjunct to existing definitions with potential to sharpen triage, timing of nephrology consultation, and dialysis planning. Prospective implementation studies—ideally with site-level calibration, safety monitoring, and co-primary endpoints that reflect both alert burden and patient-centered outcomes—are warranted to determine real-world effectiveness and equity. Further prospective studies integrating this approach into routine clinical workflows are needed to establish its practical utility and impact on decision-making and outcomes, including pragmatic prospective evaluations and, if feasible, cluster-based implementation trials.

## Supporting information

S1 Method Model configuration, optimization settings, and hyperparameters. (DOCX)

S1 Table Distribution of serum creatinine missingness by admission. (DOCX)

S2 Table Proportion of admissions by number of missing serum creatinine days. (DOCX)

S3 Table Baseline characteristics by serum creatinine missingness status. (DOCX)

S4 Table Incidence of kidney replacement therapy and odds ratios according to anomaly detection and acute kidney injury stage 2. (DOCX)

S5 Table Incidence of in-hospital mortality and odds ratios according to anomaly detection and acute kidney injury stage 2. (DOCX)

S6 Table Area under the receiver operating characteristic curve in complete-case admissions. (DOCX)

S7 Table Threshold-dependent classification performance of the anomaly detection for predicting kidney replacement therapy. (DOCX)

S8 Table Threshold-dependent classification performance of the anomaly detection for predicting in-hospital mortality. (DOCX)

S9 Table Area under the receiver operating characteristic curve by input window length and prediction horizon for kidney replacement therapy and in-hospital mortality. (DOCX)
