# Supplementary material for: Deep-learning time-series anomaly detection of acute kidney injury from creatinine–eGFR trajectories in the ICU
Source: PLOS Digit Health. 2026 May 13;5(5):e0001411. doi: 10.1371/journal.pdig.0001411 (PMC13170855; doi:10.1371/journal.pdig.0001411)
Supplement: S1 Method — (DOCX) [file pdig.0001411.s001.docx]

**Supplementary Methods**

Architecture: We implemented an Anomaly Transformer with window size T = 7, two input channels (creatinine and eGFR), and two reconstruction outputs. The embedding stack comprised a 1D convolutional token/value embedding and sinusoidal positional embedding, followed by anomaly-attention encoder blocks with residual connections, layer normalization, and position-wise feed-forward layers. Unless otherwise stated, dropout = 0.0 and activation = GELU.

Hyperparameters: d_model = 2048; n_heads = 12; e_layers = 1; d_ff = 2048; batch size = 128; maximum epochs = 500; early-stopping patience = 10; optimizer = Adam with initial learning rate 1e−3; reconstruction loss = MSE; discrepancy weight k = 0.5; random seed = 2 (NumPy, Python random, PyTorch).

Objective: Reconstruction used mean-squared error ${(L}_{rec})$ and symmetric KL terms between series- and prior-association distributions: ($L\_symKL$). The training step followed two surrogate losses:

$$L1 = L\_rec - k \cdot L\_symKL; L2 = L\_rec + k \cdot L\_symKL$$

Validation and early stopping: At each epoch, validation computed the same surrogates ($L1$, $L2$). Early stopping monitored non-improvement in either loss with patience = 10 epochs.

Learning-rate schedule: ${lr}_{e}$*=*${0.5}^{(e-1)}$*×*${10}^{-3}$
