# Supplementary material for: Deep-learning time-series anomaly detection of acute kidney injury from creatinine–eGFR trajectories in the ICU
Source: PLOS Digit Health. 2026 May 13;5(5):e0001411. doi: 10.1371/journal.pdig.0001411 (PMC13170855; doi:10.1371/journal.pdig.0001411)
Supplement: S1 Table — (DOCX) [file pdig.0001411.s002.docx]

S1 Table. Distribution of serum creatinine missingness by admission

| **Dataset** | **Missing days per admission, mean ± SD** | **Median days per admission (IQR)** | **Admissions with ≥1 missing value (%)** |
| --- | --- | --- | --- |
| MIMIC-III and IV  (N = 81876) | 0.61 ± 0.93 | 1 (0,1) | 50.27 |
| eICU-CRD  (N = 140237) | 0.55 ± 1.42 | 0 (0,1) | 37.65 |

Abbreviation: SD, standard deviation; IQR, interquartile range; MIMIC, Medical Information Mart for Intensive Care; eICU-CRD, electronic Intensive Care Unit Collaborative Research Database.
