# Supplementary material for: Deep-learning time-series anomaly detection of acute kidney injury from creatinine–eGFR trajectories in the ICU
Source: PLOS Digit Health. 2026 May 13;5(5):e0001411. doi: 10.1371/journal.pdig.0001411 (PMC13170855; doi:10.1371/journal.pdig.0001411)
Supplement: S2 Table — (DOCX) [file pdig.0001411.s003.docx]

S2 Table. Proportion of admissions by number of missing serum creatinine days

| **Missing days per admission** | **MIMIC III and IV (%)** | **eICU-CRD (%)** |
| --- | --- | --- |
| 0 | 49.7 | 62.4 |
| 1 | 43.5 | 28.1 |
| 2 | 5.1 | 6.3 |
| 3 | 0.9 | 1.8 |
| 4 | 0.4 | 0.7 |
| 5 | 0.1 | 0.3 |
| 6 | 0.1 | 0.1 |
| 7 | 0.1 | 0.1 |
| 8 | <0.1 | 0.1 |
| >9 | <0.1 | <0.1 |

Abbreviation: MIMIC, Medical Information Mart for Intensive Care; eICU-CRD, electronic Intensive Care Unit Collaborative Research Database.
