# Supplementary material for: Deep-learning time-series anomaly detection of acute kidney injury from creatinine–eGFR trajectories in the ICU
Source: PLOS Digit Health. 2026 May 13;5(5):e0001411. doi: 10.1371/journal.pdig.0001411 (PMC13170855; doi:10.1371/journal.pdig.0001411)
Supplement: S3 Table — (DOCX) [file pdig.0001411.s004.docx]

S3 Table. Baseline characteristics by serum creatinine missingness status

| **Variable** | **MIMIC III and IV** | | | **eICU-CRD** | | |
| --- | --- | --- | --- | --- | --- | --- |
|  | **Admissions without missing day** | **Admissions with ≥1 missing day** | **SMD** | **Admissions without missing day** | **Admissions with ≥1 missing day** | **SMD** |
| Male (%) | 55.9 | 56.7 | 0.02 | 53.9 | 53.9 | <0.01 |
| Age (years) | 65 (53,76) | 66 (55,77) | 0.05 | 65 (53,77) | 65 (53,77) | -0.01 |
| Minimum creatinine (mg/dL) | 0.80 (0.60,1.15) | 0.73 (0.55,1.00) | -0.20 | 0.82 (0.61,1.16) | 0.78 (0.59,1.04) | -0.16 |
| Maximum creatinine (mg/dL) | 1.13 (0.86,1.80) | 1.09 (0.85,1.54) | -0.18 | 1.20 (0.90,1.84) | 1.09 (0.85,1.53) | -0.19 |
| Kidney replacement therapy before discharge (%) | 5.6 | 0.4 | -0.31 | 2.7 | 0.3 | -0.19 |
| In-hospital mortality (%) | 6.7 | 7.1 | 0.02 | 6.1 | 1.4 | 0.19 |

Values are presented as % or median (interquartile range).

Abbreviation: MIMIC, Medical Information Mart for Intensive Care; eICU-CRD, electronic Intensive Care Unit Collaborative Research Database; SMD, standardized mean difference.
