# Supplementary material for: Deep-learning time-series anomaly detection of acute kidney injury from creatinine–eGFR trajectories in the ICU
Source: PLOS Digit Health. 2026 May 13;5(5):e0001411. doi: 10.1371/journal.pdig.0001411 (PMC13170855; doi:10.1371/journal.pdig.0001411)
Supplement: S4 Table — (DOCX) [file pdig.0001411.s005.docx]

S4 Table. Incidence of kidney replacement therapy and odds ratios according to anomaly detection and acute kidney injury stage 2

| **Dataset** | **Outcome time horizon (hours)** | **Risk stratum (Anomaly rule / AKI stage 2)** | **No. of windows** | **No. of outcome events** | **Event rate (%)** | **Odds ratio** | **p value** |
| --- | --- | --- | --- | --- | --- | --- | --- |
| Internal validation  (test data in MIMIC III/IV) | 24 | Anomaly− / AKI stage 2− | 53831 | 185 | 0.34 | Reference |  |
|  | 24 | Anomaly+ / AKI stage 2− | 1256 | 52 | 4.14 | 12.52 (9.16, 17.13) | <0.001 |
|  | 24 | Anomaly- / AKI stage 2+ | 1168 | 32 | 2.74 | 8.17 (5.59, 11.94) | <0.001 |
|  | 24 | Anomaly+ / AKI stage 2+ | 1000 | 91 | 9.10 | 29.03 (22.40, 37.63) | <0.001 |
|  | 48 | Anomaly− / AKI stage 2− | 53831 | 349 | 0.65 | Reference |  |
|  | 48 | Anomaly+ / AKI stage 2− | 1256 | 113 | 9.00 | 15.15 (12.16, 18.88) | <0.001 |
|  | 48 | Anomaly- / AKI stage 2+ | 1168 | 53 | 4.54 | 7.28 (5.42, 9.78) | <0.001 |
|  | 48 | Anomaly+ / AKI stage 2+ | 1000 | 170 | 17.00 | 31.39 (25.81, 38.17) | <0.001 |
|  | 72 | Anomaly− / AKI stage 2− | 53831 | 493 | 0.92 | Reference |  |
|  | 72 | Anomaly+ / AKI stage 2− | 1256 | 169 | 13.46 | 16.82 (13.98, 20.23) | <0.001 |
|  | 72 | Anomaly- / AKI stage 2+ | 1168 | 72 | 6.16 | 7.11 (5.51, 9.17) | <0.001 |
|  | 72 | Anomaly+ / AKI stage 2+ | 1000 | 221 | 22.10 | 30.69 (25.80, 36.52) | <0.001 |
|  | 96 | Anomaly− / AKI stage 2− | 53831 | 594 | 1.10 | Reference |  |
|  | 96 | Anomaly+ / AKI stage 2− | 1256 | 181 | 14.41 | 15.09 (12.64, 18.01) | <0.001 |
|  | 96 | Anomaly- / AKI stage 2+ | 1168 | 83 | 7.11 | 6.86 (5.41, 8.69) | <0.001 |
|  | 96 | Anomaly+ / AKI stage 2+ | 1000 | 248 | 24.80 | 29.56 (25.07, 34.85) | <0.001 |
| External validation  (eICU-CRD) | 24 | Anomaly− / AKI stage 2− | 469,863 | 1,943 | 0.41 | Reference |  |
|  | 24 | Anomaly+ / AKI stage 2− | 9,165 | 217 | 2.37 | 5.84 (5.07, 6.73) | <0.001 |
|  | 24 | Anomaly- / AKI stage 2+ | 8,298 | 141 | 1.70 | 4.16 (3.50, 4.95) | <0.001 |
|  | 24 | Anomaly+ / AKI stage 2+ | 7,358 | 221 | 3.00 | 7.46 (6.48, 8.59) | <0.001 |
|  | 48 | Anomaly− / AKI stage 2− | 469,863 | 2,874 | 0.61 | Reference |  |
|  | 48 | Anomaly+ / AKI stage 2− | 9,165 | 515 | 5.62 | 9.67 (8.79, 10.65) | <0.001 |
|  | 48 | Anomaly- / AKI stage 2+ | 8,298 | 233 | 2.81 | 4.69 (4.10, 5.37) | <0.001 |
|  | 48 | Anomaly+ / AKI stage 2+ | 7,358 | 485 | 6.59 | 11.47 (10.38, 12.66) | <0.001 |
|  | 72 | Anomaly− / AKI stage 2− | 469,863 | 3,475 | 0.74 | Reference |  |
|  | 72 | Anomaly+ / AKI stage 2− | 9,165 | 648 | 7.07 | 10.21 (9.36, 11.13) | <0.001 |
|  | 72 | Anomaly- / AKI stage 2+ | 8,298 | 320 | 3.86 | 5.38 (4.79, 6.05) | <0.001 |
|  | 72 | Anomaly+ / AKI stage 2+ | 7,358 | 653 | 8.87 | 13.07 (11.98, 14.26) | <0.001 |
|  | 96 | Anomaly− / AKI stage 2− | 469,863 | 3,926 | 0.84 | Reference |  |
|  | 96 | Anomaly+ / AKI stage 2− | 9,165 | 737 | 8.04 | 10.38 (9.57, 11.26) | <0.001 |
|  | 96 | Anomaly- / AKI stage 2+ | 8,298 | 372 | 4.48 | 5.57 (5.00, 6.21) | <0.001 |
|  | 96 | Anomaly+ / AKI stage 2+ | 7,358 | 762 | 10.36 | 13.71 (12.64, 14.87) | <0.001 |

Abbreviation: AKI, acute kidney injury; MIMIC, Medical Information Mart for Intensive Care; eICU-CRD, electronic Intensive Care Unit Collaborative Research Database.
