# Supplementary material for: Deep-learning time-series anomaly detection of acute kidney injury from creatinine–eGFR trajectories in the ICU
Source: PLOS Digit Health. 2026 May 13;5(5):e0001411. doi: 10.1371/journal.pdig.0001411 (PMC13170855; doi:10.1371/journal.pdig.0001411)
Supplement: S5 Table — (DOCX) [file pdig.0001411.s006.docx]

S5 Table. Incidence of in-hospital mortality and odds ratios according to anomaly detection and acute kidney injury stage 2

| **Dataset** | **Outcome time horizon (hours)** | **Risk stratum (Anomaly rule / AKI stage 2)** | **No. of windows** | **No. of outcome events** | **Event rate (%)** | **Odds ratio** | **p value** |
| --- | --- | --- | --- | --- | --- | --- | --- |
| Internal validation  (test data in MIMIC III/IV) | 24 | Anomaly− / AKI stage 2− | 53831 | 552 | 1.03 | Reference |  |
|  | 24 | Anomaly+ / AKI stage 2− | 1256 | 57 | 4.54 | 4.59 (3.47, 6.06) | <0.001 |
|  | 24 | Anomaly- / AKI stage 2+ | 1168 | 51 | 4.37 | 4.41 (3.29, 5.91) | <0.001 |
|  | 24 | Anomaly+ / AKI stage 2+ | 1000 | 69 | 6.9 | 7.15 (5.52, 9.26) | <0.001 |
|  | 48 | Anomaly− / AKI stage 2− | 53831 | 1072 | 1.99 | Reference |  |
|  | 48 | Anomaly+ / AKI stage 2− | 1256 | 102 | 8.12 | 4.35 (3.52, 5.37) | <0.001 |
|  | 48 | Anomaly- / AKI stage 2+ | 1168 | 92 | 7.88 | 4.21 (3.37, 5.25) | <0.001 |
|  | 48 | Anomaly+ / AKI stage 2+ | 1000 | 121 | 12.1 | 6.77 (5.55, 8.27) | <0.001 |
|  | 72 | Anomaly− / AKI stage 2− | 53831 | 1569 | 2.91 | Reference |  |
|  | 72 | Anomaly+ / AKI stage 2− | 1256 | 134 | 10.67 | 3.98 (3.30, 4.79) | <0.001 |
|  | 72 | Anomaly- / AKI stage 2+ | 1168 | 128 | 10.96 | 4.10 (3.39, 4.96) | <0.001 |
|  | 72 | Anomaly+ / AKI stage 2+ | 1000 | 165 | 16.5 | 6.58 (5.53, 7.84) | <0.001 |
|  | 96 | Anomaly− / AKI stage 2− | 53831 | 2000 | 3.72 | Reference |  |
|  | 96 | Anomaly+ / AKI stage 2− | 1256 | 158 | 12.58 | 3.73 (3.14, 4.43) | <0.001 |
|  | 96 | Anomaly- / AKI stage 2+ | 1168 | 162 | 13.87 | 4.17 (3.51, 4.96) | <0.001 |
|  | 96 | Anomaly+ / AKI stage 2+ | 1000 | 202 | 20.2 | 6.56 (5.59, 7.70) | <0.001 |
| External validation  (eICU-CRD) | 24 | Anomaly− / AKI stage 2− | 469906 | 5029 | 1.07 | Reference |  |
|  | 24 | Anomaly+ / AKI stage 2− | 9122 | 336 | 3.68 | 3.54 (3.16, 3.96) | <0.001 |
|  | 24 | Anomaly- / AKI stage 2+ | 8305 | 475 | 5.72 | 5.61 (5.09, 6.18) | <0.001 |
|  | 24 | Anomaly+ / AKI stage 2+ | 7351 | 470 | 6.39 | 6.31 (5.73, 6.96) | <0.001 |
|  | 48 | Anomaly− / AKI stage 2− | 469906 | 10884 | 2.32 | Reference |  |
|  | 48 | Anomaly+ / AKI stage 2− | 9122 | 749 | 8.21 | 3.77 (3.49, 4.08) | <0.001 |
|  | 48 | Anomaly- / AKI stage 2+ | 8305 | 882 | 10.62 | 5.01 (4.66, 5.39) | <0.001 |
|  | 48 | Anomaly+ / AKI stage 2+ | 7351 | 943 | 12.83 | 6.21 (5.78, 6.66) | <0.001 |
|  | 72 | Anomaly− / AKI stage 2− | 469906 | 16186 | 3.44 | Reference |  |
|  | 72 | Anomaly+ / AKI stage 2− | 9122 | 1040 | 11.4 | 3.61 (3.38, 3.85) | <0.001 |
|  | 72 | Anomaly- / AKI stage 2+ | 8305 | 1134 | 13.65 | 4.43 (4.16, 4.73) | <0.001 |
|  | 72 | Anomaly+ / AKI stage 2+ | 7351 | 1252 | 17.03 | 5.75 (5.40, 6.13) | <0.001 |
|  | 96 | Anomaly− / AKI stage 2− | 469906 | 20691 | 4.4 | Reference |  |
|  | 96 | Anomaly+ / AKI stage 2− | 9122 | 1222 | 13.4 | 3.36 (3.16, 3.57) | <0.001 |
|  | 96 | Anomaly- / AKI stage 2+ | 8305 | 1328 | 15.99 | 4.13 (3.89, 4.39) | <0.001 |
|  | 96 | Anomaly+ / AKI stage 2+ | 7351 | 1455 | 19.79 | 5.36 (5.05, 5.68) | <0.001 |

Abbreviation: AKI, acute kidney injury; MIMIC, Medical Information Mart for Intensive Care; eICU-CRD, electronic Intensive Care Unit Collaborative Research Database.
