# Supplementary material for: Deep-learning time-series anomaly detection of acute kidney injury from creatinine–eGFR trajectories in the ICU
Source: PLOS Digit Health. 2026 May 13;5(5):e0001411. doi: 10.1371/journal.pdig.0001411 (PMC13170855; doi:10.1371/journal.pdig.0001411)
Supplement: S6 Table — (DOCX) [file pdig.0001411.s007.docx]

S6 Table. Area under the receiver operating characteristic curve in complete-case admissions

| **Dataset** | **Outcome time horizon (hours)** | **AUROC for KRT** | **AUROC for in-hospital mortality** |
| --- | --- | --- | --- |
| Internal validation  (test data in MIMIC III/IV) | 24 | 0.81 | 0.73 |
|  | 48 | 0.80 | 0.69 |
|  | 72 | 0.80 | 0.67 |
|  | 96 | 0.78 | 0.66 |
| External validation  (eICU-CRD) | 24 | 0.73 | 0.70 |
|  | 48 | 0.73 | 0.68 |
|  | 72 | 0.73 | 0.66 |
|  | 96 | 0.73 | 0.65 |

Abbreviation: AUROC, area under the receiver operating characteristic curve; KRT, kidney replacement therapy; MIMIC, Medical Information Mart for Intensive Care; eICU-CRD, electronic Intensive Care Unit Collaborative Research Database.
