# Supplementary material for: Deep-learning time-series anomaly detection of acute kidney injury from creatinine–eGFR trajectories in the ICU
Source: PLOS Digit Health. 2026 May 13;5(5):e0001411. doi: 10.1371/journal.pdig.0001411 (PMC13170855; doi:10.1371/journal.pdig.0001411)
Supplement: S7 Table — (DOCX) [file pdig.0001411.s008.docx]

S7 Table. Threshold-dependent classification performance of the anomaly detection for predicting kidney replacement therapy

| **Dataset** | **Outcome time horizon (hours)** | **Anomaly threshold (%)** | **Accuracy** | **F1 score** | **Precision** | **Recall** |
| --- | --- | --- | --- | --- | --- | --- |
| Internal validation  (test data in MIMIC III/IV) | 24 | 1 | 0.99 | 0.16 | 0.13 | 0.20 |
|  | 24 | 2.5 | 0.97 | 0.15 | 0.09 | 0.36 |
|  | 24 | 10 | 0.90 | 0.07 | 0.04 | 0.60 |
|  | 48 | 1 | 0.98 | 0.21 | 0.23 | 0.19 |
|  | 48 | 2.5 | 0.97 | 0.22 | 0.16 | 0.33 |
|  | 48 | 10 | 0.90 | 0.12 | 0.07 | 0.59 |
|  | 72 | 1 | 0.98 | 0.22 | 0.30 | 0.18 |
|  | 72 | 2.5 | 0.97 | 0.26 | 0.22 | 0.32 |
|  | 72 | 10 | 0.90 | 0.16 | 0.10 | 0.58 |
|  | 96 | 1 | 0.98 | 0.22 | 0.32 | 0.16 |
|  | 96 | 2.5 | 0.97 | 0.27 | 0.24 | 0.30 |
|  | 96 | 10 | 0.90 | 0.18 | 0.11 | 0.56 |
| External validation  (eICU-CRD) | 24 | 1 | 0.98 | 0.13 | 0.17 | 0.10 |
|  | 24 | 2.5 | 0.97 | 0.17 | 0.15 | 0.19 |
|  | 24 | 10 | 0.91 | 0.12 | 0.08 | 0.37 |
|  | 48 | 1 | 0.98 | 0.13 | 0.18 | 0.10 |
|  | 48 | 2.5 | 0.97 | 0.17 | 0.15 | 0.19 |
|  | 48 | 10 | 0.91 | 0.13 | 0.08 | 0.37 |
|  | 72 | 1 | 0.98 | 0.13 | 0.18 | 0.10 |
|  | 72 | 2.5 | 0.97 | 0.17 | 0.15 | 0.19 |
|  | 72 | 10 | 0.91 | 0.13 | 0.08 | 0.37 |
|  | 96 | 1 | 0.98 | 0.13 | 0.18 | 0.10 |
|  | 96 | 2.5 | 0.97 | 0.17 | 0.15 | 0.19 |
|  | 96 | 10 | 0.91 | 0.13 | 0.08 | 0.37 |

Abbreviation: AKI, acute kidney injury; MIMIC, Medical Information Mart for Intensive Care; eICU-CRD, electronic Intensive Care Unit Collaborative Research Database.
