# Supplementary material for: Deep-learning time-series anomaly detection of acute kidney injury from creatinine–eGFR trajectories in the ICU
Source: PLOS Digit Health. 2026 May 13;5(5):e0001411. doi: 10.1371/journal.pdig.0001411 (PMC13170855; doi:10.1371/journal.pdig.0001411)
Supplement: S8 Table — (DOCX) [file pdig.0001411.s009.docx]

S8 Table. Threshold-dependent classification performance of the anomaly detection for predicting in-hospital mortality

| **Dataset** | **Outcome time horizon (hours)** | **Anomaly threshold (%)** | **Accuracy** | **F1 score** | **Precision** | **Recall** |
| --- | --- | --- | --- | --- | --- | --- |
| Internal validation  (test data in MIMIC III/IV) | 24 | 1 | 0.98 | 0.05 | 0.06 | 0.04 |
|  | 24 | 2.5 | 0.97 | 0.08 | 0.06 | 0.11 |
|  | 24 | 10 | 0.89 | 0.07 | 0.04 | 0.30 |
|  | 48 | 1 | 0.97 | 0.06 | 0.10 | 0.04 |
|  | 48 | 2.5 | 0.96 | 0.11 | 0.11 | 0.11 |
|  | 48 | 10 | 0.89 | 0.12 | 0.07 | 0.30 |
|  | 72 | 1 | 0.96 | 0.07 | 0.15 | 0.04 |
|  | 72 | 2.5 | 0.95 | 0.12 | 0.15 | 0.10 |
|  | 72 | 10 | 0.88 | 0.15 | 0.10 | 0.28 |
|  | 96 | 1 | 0.95 | 0.06 | 0.18 | 0.04 |
|  | 96 | 2.5 | 0.94 | 0.12 | 0.17 | 0.09 |
|  | 96 | 10 | 0.88 | 0.17 | 0.12 | 0.27 |
| External validation  (eICU-CRD) | 24 | 1 | 0.98 | 0.05 | 0.05 | 0.04 |
|  | 24 | 2.5 | 0.97 | 0.07 | 0.06 | 0.10 |
|  | 24 | 10 | 0.91 | 0.07 | 0.04 | 0.27 |
|  | 48 | 1 | 0.97 | 0.06 | 0.11 | 0.04 |
|  | 48 | 2.5 | 0.96 | 0.10 | 0.11 | 0.09 |
|  | 48 | 10 | 0.90 | 0.13 | 0.08 | 0.26 |
|  | 72 | 1 | 0.95 | 0.06 | 0.14 | 0.04 |
|  | 72 | 2.5 | 0.95 | 0.11 | 0.15 | 0.09 |
|  | 72 | 10 | 0.90 | 0.15 | 0.11 | 0.24 |
|  | 96 | 1 | 0.94 | 0.06 | 0.17 | 0.03 |
|  | 96 | 2.5 | 0.94 | 0.11 | 0.17 | 0.08 |
|  | 96 | 10 | 0.89 | 0.17 | 0.13 | 0.23 |

Abbreviation: MIMIC, Medical Information Mart for Intensive Care; eICU-CRD, electronic Intensive Care Unit Collaborative Research Database.
