# Supplementary material for: Deep-learning time-series anomaly detection of acute kidney injury from creatinine–eGFR trajectories in the ICU
Source: PLOS Digit Health. 2026 May 13;5(5):e0001411. doi: 10.1371/journal.pdig.0001411 (PMC13170855; doi:10.1371/journal.pdig.0001411)
Supplement: S9 Table — (DOCX) [file pdig.0001411.s010.docx]

S9 Table. Area under the receiver operating characteristic curve by input window length and prediction horizon for kidney replacement therapy and in-hospital mortality

| **Dataset** | **Outcome time horizon (hours)** | **Input window length (days)** | **AUROC for KRT** | **AUROC for in-hospital mortality** |
| --- | --- | --- | --- | --- |
| Internal validation  (test data in MIMIC III/IV) | 24 | 2 | 0.58 | 0.44 |
|  |  | 3 | 0.78 | 0.56 |
|  |  | 4 | 0.79 | 0.60 |
|  |  | 5 | 0.82 | 0.62 |
|  |  | 6 | 0.82 | 0.64 |
|  | 48 | 2 | 0.59 | 0.45 |
|  |  | 3 | 0.76 | 0.57 |
|  |  | 4 | 0.77 | 0.60 |
|  |  | 5 | 0.80 | 0.62 |
|  |  | 6 | 0.80 | 0.63 |
|  | 72 | 2 | 0.58 | 0.44 |
|  |  | 3 | 0.75 | 0.56 |
|  |  | 4 | 0.76 | 0.59 |
|  |  | 5 | 0.79 | 0.61 |
|  |  | 6 | 0.78 | 0.61 |
|  | 96 | 2 | 0.56 | 0.44 |
|  |  | 3 | 0.73 | 0.56 |
|  |  | 4 | 0.74 | 0.58 |
|  |  | 5 | 0.77 | 0.61 |
|  |  | 6 | 0.77 | 0.61 |
| External validation  (eICU-CRD) | 24 | 2 | 0.51 | 0.42 |
|  |  | 3 | 0.68 | 0.56 |
|  |  | 4 | 0.71 | 0.59 |
|  |  | 5 | 0.73 | 0.61 |
|  |  | 6 | 0.73 | 0.62 |
|  | 48 | 2 | 0.51 | 0.44 |
|  |  | 3 | 0.68 | 0.57 |
|  |  | 4 | 0.71 | 0.60 |
|  |  | 5 | 0.73 | 0.61 |
|  |  | 6 | 0.73 | 0.62 |
|  | 72 | 2 | 0.51 | 0.44 |
|  |  | 3 | 0.68 | 0.56 |
|  |  | 4 | 0.71 | 0.59 |
|  |  | 5 | 0.73 | 0.60 |
|  |  | 6 | 0.73 | 0.61 |
|  | 96 | 2 | 0.51 | 0.44 |
|  |  | 3 | 0.68 | 0.55 |
|  |  | 4 | 0.71 | 0.58 |
|  |  | 5 | 0.73 | 0.60 |
|  |  | 6 | 0.73 | 0.60 |

Abbreviation: AUROC, area under the receiver operating characteristic curve; KRT, kidney replacement therapy; MIMIC, Medical Information Mart for Intensive Care; eICU-CRD, electronic Intensive Care Unit Collaborative Research Database.
